# HabitApp: New Play Technologies in Pediatric Cancer to Improve the Psychosocial State of Patients and Caregivers

**DOI:** 10.3389/fpsyg.2020.00157

**Published:** 2020-02-07

**Authors:** Alicia Carrion-Plaza, Javier Jaen, Inmaculada Montoya-Castilla

**Affiliations:** ^1^EMINA Group, Departamento de Personalidad, Evaluación y Tratamientos Psicológico, Facultad de Psicología, University of Valencia, Valencia, Spain; ^2^ISSI-Futurelab Group, Departamento de Sistemas Informáticos y Computación, Universitat Politècnica de València, Valencia, Spain

**Keywords:** pediatric, games, cancer, caregiver, psychosocial, intervention

## Abstract

Childhood cancer involves long periods of hospitalization that trigger emotions such as fear or sadness. Previous research has studied the positive effects of technology games on improving the hospitalization experience, but most do not focus on caregivers and none allow interaction with the real time observation of a zoo. The present study evaluates the impact of HabitApp and assesses the short-term impact on the psychosocial state of patients and caregivers in order to improve the hospitalization experience. The participants in this study were 39 patients plus 39 caregivers. A quantitative analysis revealed a significant improvement in patient’s and caregiver’s psychosocial factors from the pre-play period to 10 min play time, and a significant interaction effect between the psychological state and the impact of HabitApp. The children with highest levels of depression obtained most benefit from the positive effects. A qualitative analysis brought out four themes: physical state, emotional state, social interaction, and hospital routines. Using a user experience questionnaire the patients and caregiver reported high satisfaction rates with the app use. These results confirm that it is important to continue studying this type of technology in order to develop better interventions to be included in an integral approach to this pathology considering caregivers into pediatric oncology patients’ play therapy.

## Introduction

Currently, childhood cancer has a high incidence rate. Internationally, 156 new cases per million children aged from 0 to 19 are diagnosed each year (Steliarova-Foucher et al., 2017). In Spain, 931 new cases of cancer were detected in children between 0 and 14 years old in 2017 only. One hundred of these cases were in the Comunidad Valenciana, where the study was carried out (Asociación Española Contra el Cáncer [(AECC], 2018). However, advances in treatment have increased the survival rate in recent years. The ratings indicate that 80% of patients aged 0–14 years survive at least 5 years from the date of diagnosis (Peris et al., 2017).

As a result, in many cases, cancer is considered as a chronic disease requiring long-term treatments with associated secondary effects that have to be controlled (Ibáñez and Baquero, 2009).

Several empirical studies have determined that the disease and the side effects of different types of treatment (chemotherapy, radiation therapy and bone marrow transplantation) can have a negative impact on the lives of children and their families (Barahona et al., 2009). Long periods of hospitalization and repeated admissions trigger different emotions like fear, anger or sadness, which may have a negative impact on the children’s life and their ability to adapt to the disease (Méndez et al., 2004). Therefore, children with cancer are more vulnerable to pain, social isolation, depression, and low school performance (Schultz et al., 2007), also caregivers are more vulnerable to anxiety, depression and physical illness as a consequence of psychological stress and fear associated with oncological illness and hospitalization (Wechsler et al., 2014).

Caregivers often have more adaptability problems related with medical procedures than those presented by their pediatric patients (Pinquart, 2017). In this regard, there is evidence of emotion transfer between parents and children during hospitalization, with more intense reactions and symptoms in the patients, such as nausea or vomiting, in those cases where the caregiver shows a high level of anxiety (Fernández-Castillo and López-Naranjo, 2006). For this reason, it is necessary to respond to this pathology comprehensively by developing multidisciplinary interventions with the aim of improving the patients and caregiver’s quality of life (Méndez et al., 2004).

Nowadays, psychosocial interventions developed for childhood cancer are focused on improving the coping skills of patients and families by increasing understanding of the disease and providing support groups (Suzuki and Kato, 2003), as well as by improving the quality of life and psychological wellbeing by reducing emotional problems (Ibáñez and Baquero, 2009). These interventions involve behaviors such as listening, showing affection, showing interest, guiding, or expressing acceptance, in order to fulfill an emotional, material or informational function that may help to reduce anxiety caused by the diagnosis and treatment, to reduce feelings of isolation and abandonment, and to contribute to a better quality of life during the illness process (Bárez et al., 2003).

Play therapies have also been used and recommended as an essential part of children’s hospital care (Montoya and Barrón, 2001). Play helps to deal with the psychological problems of children with cancer because it promotes their intellectual, emotional and social development, allowing them to find motivations and satisfactions that improve their wellbeing (Romagosa, 2005).

There is evidence that play therapy has positive effects on children with cancer by inducing relaxation, distraction and improvements in patient socialization (Ortigosa et al., 2009; Lima and Santos, 2015). These results can improve the symptoms associated with treatments (Salas et al., 2004). It has also been shown to be helpful for expressing positive emotions in these children (Paixão et al., 2016), which is a key aspect of rehabilitation found in recent studies on resilience in childhood cancer (Pintado and Cruz, 2017). The other main benefit of this kind of intervention is the enhancement of children’s adherence to the treatment, for example chemotherapy treatment (Artilheiro et al., 2011) and the reduction of the associated levels of anxiety (Sajjad et al., 2014).

Traditionally, the interventions used for this purpose have been art therapy (Favara-Scacco et al., 2001), music therapy (Barrera et al., 2002), and laughter therapy (Sánchez et al., 2009). The instruments used were books, papers and crayons for different activities, according to the age range of the children (Pedrosa et al., 2007).

Innovative play therapy approaches use new information and communication technologies (ICT), given their wide acceptance by today’s youth and their greater degree of immersion (Duffy et al., 2005). Different research studies have proven that the use of technology as a play therapy tool may reduce symptoms such as nausea associated with chemotherapy, provide distraction in invasive procedures, such as venepuncture, encourage physical rehabilitation of children in chronic, neurological or traumatic diseases and increase adaptation to the disease (Li et al., 2011; Caldwell et al., 2013; Sajjad et al., 2014).

In addition to these clinical uses, technologies have also been used in childhood cancer to facilitate contact with the outside world, taking into account the isolation conditions that characterize some phases of this disease (Shama and Lucchetta, 2007; Liu et al., 2015). The main objective of these studies was to facilitate communication between patients and caregivers, their friends or family outside the hospital, placing the patients in a context which is external to the source of stress.

All the above technological interventions are designed for a single pediatric patient, with caregivers either as a secondary participant or left out of the activity (Nilsson et al., 2009; Gerling et al., 2011; Caldwell et al., 2013; Sajjad et al., 2014; Lima and Santos, 2015). Therefore, most play interventions do not consider caregivers as a direct target together with children, although some studies show that hospitalized children prefer to play with their parents in their room rather than alone or with volunteers in hospital wards (Sánchez, 2011). It is important to include parents in the therapeutic process to facilitate positive emotional bonds between them (Bratton et al., 2005). Moreover, most technological interventions that consider placing the patients in an external context to alleviate the stress produced by long-term hospital confinement do so by implementing infrastructures for children to communicate with relatives and friends who may not always be present. This reduces the opportunities to use these strategies during hospitalization and may also increase the sense of isolation when hospitalized children are unable to communicate with their loved ones.

In this context, and as we will discuss in detail in Section “Related Works,” we can state that there is a lack of studies on the positive emotional impact of combining all these factors: a technological play therapy solution that places the patients in contact with an external context which is permanently present and involves not only the patient but also the caregivers as potential target users. This paper thus proposes HabitApp, a technological infrastructure designed to cope with these requirements, and evaluates its short-term effectiveness as a play therapy intervention in the context of childhood cancer. The specific objectives were to determine whether the patients and caregivers’ psychosocial state improves during game play, to assess as a *post hoc* evaluation whether their psychological state influences the impact of the intervention and finally to obtain evidence about the user experience with the technology.

## Related Works

### Impact of Technological Game Interventions on Hospital Experience and Adaptation

Several works have studied the deployment of technologies for play in the context of pediatric oncology hospitalization. Some of these studies only evaluated user satisfaction and experience, as in the case of ZORA, Bers et al. (2001), who proposed a virtual environment to help patients explore identity issues in which pediatric patients could build virtual rooms, chat with others in real time through an avatar and send messages or stories to each other. The results showed that this was a safe and context-appropriate game which users found satisfactory. For the same purpose, Fels et al. (2003) studied the usability and usefulness of a technological solution for connecting with the outside world through a BlackBerry PDA. The results indicated that it is most useful for short-stay hospitalized children and young people. Gerling et al. (2011) presented a virtual environment in which children could create a social support network, with the aim of helping patients to maintain contact with their peers when face-to-face contact is impossible. The results showed positive feedback and acceptance. Videogames have also been used to encourage collaborative play between peers. Fuchslocher et al. (2011) evaluated the impact of the videogame “Adventures in Sophoria” with the aim of facilitating intercommunication among adolescents during cancer treatment and found high levels of game acceptance.

Another interesting work with the aim of fostering communication between patients and caregivers is that of Quintinilla et al. (2014), who assessed the impact of an ecological park inside a hospital with many technological play elements. The results of this experience showed a high level of acceptance and satisfaction. Barbosa et al. (2014) assessed the use of technologies such as educational tablets and games to strengthen curricular activities for children and adolescents undergoing oncology, to prevent them from falling behind in their school work. The results showed excellent acceptance from patients and caregivers.

Other studies assessed the impact of the intervention to improve adaptation to hospital experience in pediatric oncology, including a measurement methodology.

As previously mentioned, these works focus mainly on creating communities in which patients and caregivers can find support from their peers and family members online. Farnham et al. (2002) evaluated HutchWorld, a virtual community for bone marrow transplant patients and their caregivers, which provided social and informational support. This study found that the impact of internet access and use of HutchWorld improved life satisfaction and provided adequate social support after the transplant procedure.

Similarly, Shama and Lucchetta (2007) tested an ICT program to connect adolescent leukemia patients with each other and reconnect them with their friends through involvement as part of “normal” events. The results proved that adolescents could improve their psychosocial skills.

Another research area of technological play intervention has traditionally focused on helping patients and caregivers to understand their diseases and improve treatment adherence. In this line, the work of Brokstein et al. (2002) is an example, where the objective was to facilitate adjustment during the initial diagnosis and treatment, serving as a preparation for medical interventions and pediatric oncology. This intervention increased the children’s and family members’ sense of control over cancer. Dragone et al. (2002) presented a virtual environment named “A Space Adventure” and concluded that the feeling of control was increased and high game satisfaction was achieved.

Lastly, with the aim of improving adherence to self-care and medication intake, the work of Kato et al. (2008) evaluated the benefits of videogames and also obtained satisfactory results.

Considering the shortcomings found regarding the use of play therapy in a children’s cancer wards, our first research question is:

RQA: What psychosocial impact does HabitApp as a game intervention have on patients?

**Hypothesis 1**: Our proposed technological game intervention will have a positive impact on the psychosocial state of the patients, incrementing the levels of affection, physical activity, social interaction, interest, and satisfaction, and decreasing the levels of somatic complains and nervousness after the intervention.

### Impact of Technological Game Intervention on Caregivers as Targets of Technological Game Intervention

Information and communication technologies play-therapy interventions have often included caregivers as target users together with pediatric patients. In this respect, the works of Brokstein et al. (2002) and Barbosa et al. (2014) analyzed the impact of educational activities supported by ICTs, such as co-localized and collaborative games, obtaining high levels of interaction between patients and caregivers. In this same line, the ecological park in the hospital described by Quintinilla et al. (2014) also involved patients and caregivers in co-localized and collaborative activities. The results of this study show that 86% of the caregivers were very interested in having their children participate in activities and 96% in having the service continue in the hospital, although they considered safety and cost the most important factors.

Other studies, however, consider caregivers in the intervention but are not designed to facilitate interaction between them and patients. For example, in Farnham et al. (2002) a virtual community is proposed for patients and caregivers, but conceived as an individual resource that each stakeholder uses independently, placing interaction in an online activity with peers.

This review has revealed that there is a lack of studies that consider caregivers as recipients of technology game interventions and assess patient-caregiver interaction. This motivates our second research question:

RQB: What psychosocial impact does HabitApp as a game intervention have on caregivers?

**Hypothesis 2**: Our proposed technological game intervention will have a positive impact on the psychosocial state of caregivers, incrementing the levels of affection, proximity, interest, satisfaction and emotional reaction, and decreasing the levels of nervousness.

### Effect of Psychological State on the Impact of Technological Game Intervention

Psychological symptoms triggered by the disease could affect patients and caregivers’ reactions to hospital interventions, and some studies have taken these factors into account. However, most studies just evaluate the impact of the intervention on the psychological state without taking into account how the starting psychological state affects the effectiveness of the intervention. For instance, Li et al. (2011) considered this aspect and assessed the symptoms of anxiety and depression before and after a video-game intervention. The results confirmed that children reported statistically significant lower levels of depression after the intervention than those who did not play a game, although there were no significant differences in anxiety levels. Sajjad et al. (2014) followed a similar approach and found that the intervention significantly improved symptoms of anxiety and depression in the experimental group with respect to a control group.

Immersion technologies such as virtual reality (VR) have also been used for this purpose. Windich-Biermeier et al. (2007) and Nilsson et al. (2009) proposed VR interventions to improve patient experience in the context of painful medical procedures and assessed the fear and stress suffered in both a control and an experimental group. The results showed that VR was effective in reducing fear and stress during painful procedures such as venepuncture. Given the lack of studies that consider how the effectiveness of the game intervention is affected by the starting psychological state of the players our third research question will investigate this issue:

RQC: What influence does the psychological state of the player have on the psychosocial impact of HabitApp?

**Hypothesis 3**: The positive psychosocial impact of our proposed technological game intervention will be higher for the patients (mood and depression) and caregivers (mood, depression, anxiety, somatic complaints and wellbeing) with a psychological negative state (low rating in happiness and wellbeing, and higher rating in sadness, anger, fear, depression, anxiety and somatic complaints).

The review of the above-cited studies (see Table 1) shows that there are play therapy technological solutions in pediatric oncology that provide good play experience as well as the acceptance of the technological resource and also have a positive impact on psychosocial factors. Unfortunately, we found no studies that consider the caregiver as a recipient of the intervention and assessed his/her psychological state in the context of a collaborative task to facilitate patient/caregiver interaction in a context outside the hospital environment, which is the main focus of the present work.

**TABLE 1:** Related works on technological game interventions in childhood cancer.

Author(s)	Age range	Pathology or procedure	Carer interaction	Activity	Purpose	Object of study
Barbosa et al., 2014	8–16	Cancer	Yes	Co-located	Education	User experience
Bers, 2009	11–15	Cancer: bone marrow transplant	No	Online	Socialization	User experience
Brokstein et al., 2002	10–17	Cancer: leukemia or lymphoma	Yes	Co-located	Education	Control feeling
Dragone et al., 2002	4–11	Cancer: leukemia	No	Individual	Education	User experience and control feeling
Farnham et al., 2002	≥18	Cancer	Yes	Online	Socialization	Social adjustment and life satisfaction.
Fels et al., 2003	12–18	Cancer and others	No	Online	Socialization	Psycho-emotional adjustment
Fuchslocher et al., 2011	n/a	Cancer	No	Online	Distraction and socialization	User experience
Gerling et al., 2011	7–19	Cancer	No	Online	Education	User experience
Kato et al., 2008	13–23	Cancer	No	Individual	Motivation	Self-care and treatment adherence
Li et al., 2011	8–16	Cancer	No	Co-located	Motivation	Anxiety and depression
Nilsson et al., 2009	5–18	Cancer: venepuncture	No	Individual	Distraction	Pain and distress
Quintinilla et al., 2014	5–14	Cancer	Yes	Co-located	Distraction and socialization	User experience
Sajjad et al., 2014	10–14	Cancer	No	Individual	Motivation	Anxiety and depression
Windich-Biermeier et al., 2007	5–18	Cancer: venepuncture	No	Individual	Distraction	Pain, fear and distress
Shama and Lucchetta, 2007	Teenagers	Cancer	No	Online	Communication	Positive affect

## Habit App: a Multimedia Application for Play Therapy

**FIGURE 1:**
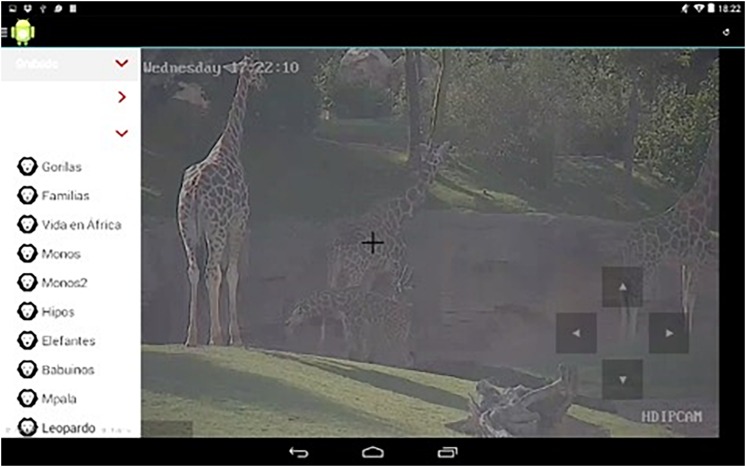
Instance of HabitApp showing a list of streams. Copyright © 2019, Polytechnic University of Valencia, Dr. J. Jaén.

**FIGURE 2:**
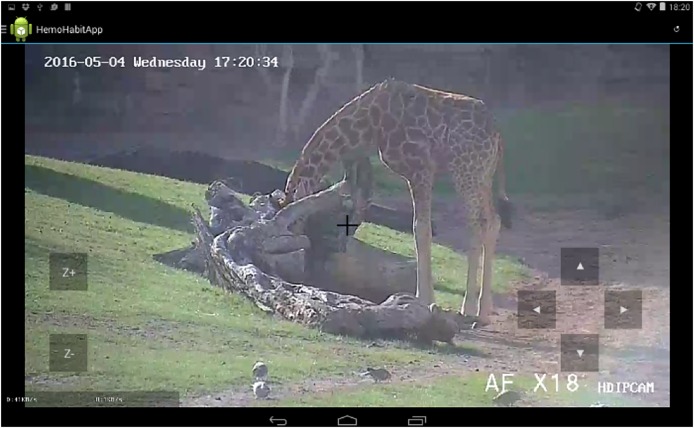
Instance of HabitApp with all control buttons. On the left, zoom-in/zoom-out; direction pointers on the right. Copyright © 2019, Polytechnic University of Valencia, Dr. J. Jaén.

To validate the previous hypotheses, together with some hospital teachers we designed the HabitApp technological play therapy solution, which puts the users in contact with a permanently present external context and involves not only the patients themselves but also caregivers as potential target users. The advantages of HabitApp with respect to previous multimedia applications are that it is designed to allow a multiuser collaborative experience, it involves animals which has been identified in the literature as a motivational activity for children, it allows children to share multimedia content related to observational activities and it provides automatic image-recognition features for children to obtain additional information about what it is being displayed on the screen (see a movie sample of HabitApp in use showing these features in HabitApp). Like Zootopia (Akabane et al., 2010), HabitApp (see Figures 1, 2) is a mobile application developed by the authors of this work that allows the observation of animals in their own habitats all over the world. In this case, the users can select the habitat they want to observe from a sliding menu on the left (see Figure 1). A live video stream is then displayed, showing the animal’s habitat. HabitApp can handle a virtually unlimited number of streams, available from the categories in the menu on the left. Currently, the application lets you choose from multiple Explore cams, which record animals in different places around the world, using either live or pre-recorded streams, and also from cameras placed at the local Bioparc zoo of Valencia, that allowed us place the cameras into the zoo. Permission was obtained to use Bioparc’s pictures and images for the present work. Pan-Tilt-Zoom (PTZ) cameras are used, which can be controlled by the application to offer a richer experience to the users. Figure 2 shows the command buttons used to control camera direction and zoom. Several users can watch video streams on their own devices at the same time, but since only one camera is available for PTZ control, only one user at a time can have the command buttons on screen. Any user can dynamically assign the control of the camera to any other connected user.

**FIGURE 3:**
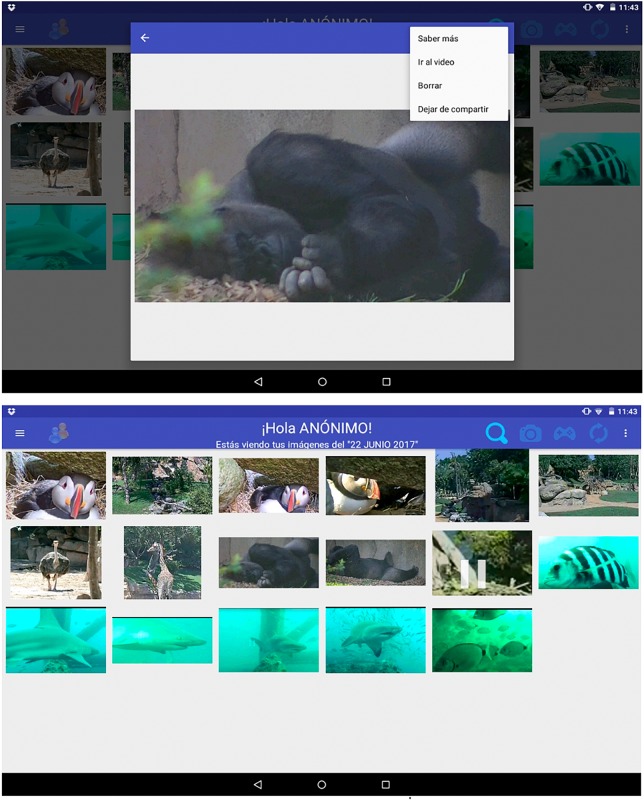
Examples of captured images and a personal album. Copyright © 2019, Polytechnic University of Valencia, Dr. J. Jaén.

Users can also capture frames from the video streams to build their own picture albums and the captured images can be published to make them available to other hospitalized children (see Figure 3).

**FIGURE 4:**
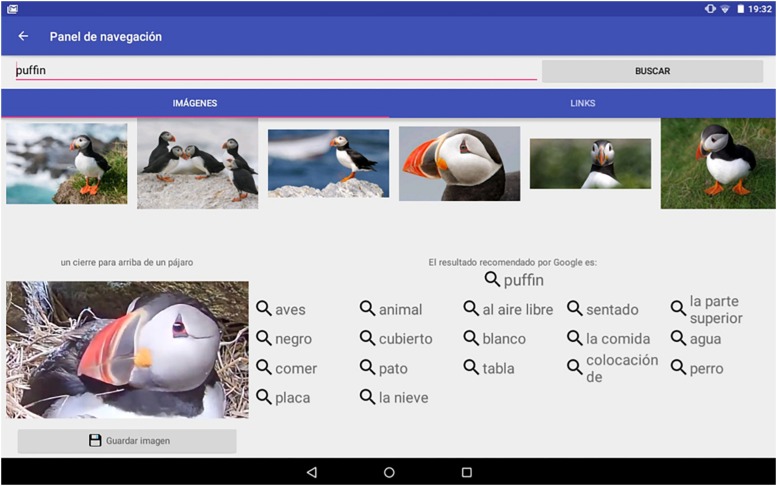
Example of query related to a captured image. Copyright © 2019, Polytechnic University of Valencia, Dr. J. Jaén.

Captured images can also be used as queries to request similar images from the system or even to ask for descriptions of what is shown on the screen (by means of image analysis algorithms) to enrich the children’s observation activities (see Figure 4).

## Materials and Methods

The overall objective of this study was to assess the impact of an intervention with HabitApp in pediatric oncology patients and their caregivers in the Pediatric Oncology Unit of the Polytechnic and University Hospital La Fe in Valencia (Spain). A transversal study was carried out to achieve this objective.

### Participants

The study was conducted on 39 oncology patients and 39 caregivers. 64% (*n* = 25) of the patients were male, with an age range of 1–16 years old (*M* = 7.18 years; *SD* = 4.5 years). The caregivers were mostly women, with 61% (*n* = 24) in an age range of 30–64 years old (*M* = 40.9 years; *SD* = 7.1). This sample used the HabitApp tool and an *ad hoc* observational assessment was conducted.

Psychological evaluation by self-reports could not be performed on all the participants. Patients over 8 years old could respond to self-reports, as indicated in the instrument application guidelines. The subsample consisted of 13 patients, 33% of the sample, 9 boys and 4 girls, (*M* = 11.92 years; *SD* = 2.8). The sample of caregivers was also reduced, due to experimental mortality, 27 caregivers (69%) answered the questionnaires, of whom 16 were women and 11 were men (*M* = 41.2 years, *SD* = 6.8).

The participants were selected considering the following inclusion criteria: patients hospitalized in the Hemato-oncological Unit of the Polytechnic and University Hospital La Fe Hospital, and aged between one and sixteen years. The exclusion criteria were: not to be in the diagnostic or terminal phases of the disease or any other patients’ clinical status that the clinical staff considers unsuitable for participation. The main caregiver was the person who spends most time caring for the patient in hospital.

### Instruments

The data were collected through an *ad hoc* observational scale that evaluates psychosocial variables, a self-report test battery that evaluates psychological variables, and a questionnaire that evaluates user experience.

#### Observational Scale: Psychosocial Variables

An observational *ad hoc* measurement scale was used to measure the psychosocial effect. This scale analyzed seven dimensions from the patient’s behavior (*affection, nervousness, somatic complaints, physical activity* and *social interaction*, *interest in play* and *satisfaction)* and six from the caregiver’s behavior’ (*affection, nervousness, proximity to the child*, and *reactions to the child’s emotions, interest in play* and *satisfaction)*, rated them with a score between 0–3, as detailed in the Supplementary Material S1.

All these dimensions were evaluated three times in each session: at the beginning (pre-play), mid-session (10 min playing [10′ playing]) and at the end (20 min playing [20′playing]), by two independent observers.

The use of this *ad hoc* scale was motivated because a lack of validated observational scales was observed for the evaluation of the behavior (physical, emotional and psychosocial) manifested by children aged 0–6 years and adults in difficult contexts, such as continuous interaction or a complex clinical state in the hospital’s environment. For this purpose, an initial non-systematic observation was made over 1 month, focused on hospital life, natural interaction between patient and caregiver, and the psychosocial factors involved. The work of Montoya-Castilla (2002) and Artilheiro et al. (2011) was also used as an example in developing the observational scale. The observed behavior generated a group of study psychosocial variables. At the same time, we constructed an evaluation scale from 0 to 3, in which 0 indicates the most negative dimension of this factor and 3 most positive. A reliability study was conducted included 58 patients (*M* = 7.00 years; *SD* = 3.28 years; 33% girls) and 22 caregivers (*M* = 39.00 years; *SD* = 5.23 years; 73% women). The observation was carried out in the Pediatric and Hemato-oncological Unit of the Hospital Universitari i Politècnic La Fe in Valencia. For the analysis of the data, the Kappa interobserver concordance index was calculated (Fleiss, 1981). The results showed very good inter-judge concordance for the features Nervousness, Physical Activity and Somatic Complaints (*K* = 0.94; 0.87; 0.90); and good in the features Affection, Interaction and Interest (*K* = 0.65; 0.75; 0.77); In caregivers the concordance was very good for Nervousness and Emotional Reaction (*K* = 0.90; 0.86); good for the Proximity and Interest trait (*K* = 0.67; 0.68); and moderate for the Affect trait (*K* = 0.60).

In order to complement the observational study, this scale included a section to add comment and notes of qualitative information about significant events that occurred during the gaming session, considered as those that had an impact on the physical or emotional state of the child or patient, or on social interaction and routines.

#### Test Battery: Psychological Variables

The psychological variables studied in the patients were: *mood* and *depression*. The psychological variables studied in the caregivers were *mood*, *depression*, *anxiety*, *somatic complaints* and *well-being*.

In patients, *mood* was assessed with the Mood Scale (MOOD) (Plumed et al., 2013), which measures the frequency of four moods: fear, sadness, happiness, and anger, with a consistency of α between 0.69 and 0.78, and *depression* was assessed with the State-Trait Depression Inventory (IDER) (Buela and Agudelo, 2008), which measures dysthymia, understood as the negative effect at the time of assessment, with good internal consistence of α of between 0.71 and 0.86.

In caregivers, *mood* and *depression* were assessed by the means cited above. Additionally, a*nxiety* was assessed by the State-Trait Anxiety Inventory (STAI) (Spielberger et al., 1994), which measures anxiety “right now” with an internal consistency of α = 0.93. *Somatic complaints* were evaluated by the Somatic Complaints List (SCL) (Plumed et al., 2015) focusing on frequency according to the complaints of pain experienced. This scale presented an internal consistency of α = 0.78, and *well-being* was assessed using the Positive and Negative Experience Scale (SPANE) (Diener et al., 2010), which measures the presence of positive and negative experiences, with an internal consistency of α of between 0.81 and 0.89.

#### Questionnaire: User Experience

User experience was evaluated through a questionnaire composed of items formulated as statements with a Likert-type response scale of five alternatives (1 = strongly disagree, 2 = disagree, 3 = undecided, 4 = agree, 5 = strongly agree, for adults, and 1 = nothing, 2 = little, 3 = something, 4 = quite a lot, 5 = very much, for children). The children’s questionnaire consisted of nine items and evaluated four dimensions: satisfaction with HabitApp (items 1, 4, 5, and 6); adequacy to context (item 2); perceived difficulty of use (items 3) and HabitApp’s future possibilities (items 7, 8, and 9). Inverted items were used to avoid effect of acquiescence (items 5 and 3). Nine items were included in the carer questionnaire and five dimensions were evaluated: their child’s satisfaction (items 1, 4, 5, and 6); his or her satisfaction (item 8); adequacy for context (item 2); difficulty of use (item 3) and HabitApp’s future possibilities (items 7 and 9). Item 6 was inverted (see detail in Supplementary Material S2).

### Equipment

Two 10-inch Android tablets and a wireless router were used to ensure proper internet connectivity.

### Procedure

The study was carried out in the Pediatrics Unit of the Polytechnic and University Hospital La Fe in Valencia from February to May 2017. To answer our research questions, a controlled study was designed consisting of play sessions with HabitApp in the patients’ rooms, 30 min sessions during 3 weeks, and an evaluation before, during and after the technological game. The ethical approval was given by the ethical commitment of the Hospital.

The procedure followed by the researchers involved several phases every day. First of all, the researchers attended the morning meeting of the medical team to establish recruitment criteria according to the patients’ clinical status. Next, the researchers presented the study and game tool to selected families for intervention, this included giving them information about the voluntary nature of participating and the anonymous processing of the data collected. When the family agreed to participate in the study, the written and informed consent was obtained from all adult participants and from the parents of all non-adult participants, and the researchers collected the patients and caregivers’ questionnaires on sociodemographic variables and psychological scales assessment.

The researchers then provided the tablets with HabitApp installed to the patient and the main caregiver, with a short introduction and training in its use. They were then given 30 min of free use with the possibility of stopping the activity at any time. During this play time two persons (a researcher and an external observer) performed a systematic observation. In order to make sure that the researcher was not inducing a personal bias an interrater agreement Kappa statistic was evaluated to detect discrepancies in the evaluations. The systematic scale of observation was completed in the following stages: (1) just before giving them the tool; (2) 10 min into the game; and (3) after 20 min of play using the observation template. They also included a description such as any specific comments or behavior as qualitative data. The final stage was the user experience questionnaire.

### Analysis

Statistical analyses were performed through the SPSS Statistical Package (V.21). ANOVA with repeated measures (RM) was used to assess the main effects in the psychosocial through the three evaluation times (pre play, 10 min playing, and 20 min playing) in the patients (affection, physical activity, social interaction, interest, satisfaction, somatic complaint, and nervousness) and caregivers (affection, proximity, interest, satisfaction, nervousness, and emotional reaction).

As a *post hoc* study, a mixed ANOVA 2 × 2 analysis was conducted to evaluate the interaction effect between these psychosocial variables and the psychological variables (patient’s fear, sadness, happiness, anger, or depression; and caregiver’s fear, sadness, happiness, anger, depression, anxiety, somatics complaints or wellbeing). The participants were divide in two group according to the *Z*-scores of the psychological variables, obtained above and below average groups (low and high levels of the variables rates).

Finally, the qualitative data related to significant events occurred during the use of HabitApp were analyzed using the grounded theory (Glaser and Strauss, 1999), analyzed by themes through in-depth inductive coding, and thematic mapping processes (Meier et al., 2007). A semantic analysis, in which the focus was data content (rather than underlying assumptions) and interpretation involved identifying the significance and implications of themes and constituent data in the context of the existing knowledge (Braun and Clarke, 2006). The key themes reflected important aspects of the HabitApp experience.

## Results

### Hypothesis 1

Our proposed technological game intervention will have a positive impact on the psychosocial state of the patients, incrementing the levels of affection, physical activity, social interaction, interest, and satisfaction, and decreasing the levels of somatic complains and nervousness after the intervention.

The results were partially in line with the formulated hypothesis. The analysis of the observational scale score by RM ANOVA yielded a significant main effect of time, suggesting a significant improvement in the children’s psychosocial factors *affection, physical activity, social interaction, interest* and *satisfaction* from the three different stages. There were no significant differences in the rating of these factors between Stages 1 and 2, while there were no significant differences in the *somatic complaints* and *nervousness* factors from all the stages (see Table 2). Neither were any significant differences found by age sex.

**TABLE 2:** *ANOVA* of psychosocial factors and observation times in patient’s ratings.

	Pre playing	10′ playing	20′ playing	Time						Result
					
	*M (SD)*	*M (SD)*	*M (SD)*	*df*	*F*	*p*	η^2^	95% Confidence interval		
									
								Lower bound	Upper bound	
Affection	1.03 (0.74)	2.15 (0.75)	2.21 (0.66)	2	59.8	0.000^∗^	0.61	1.61	1.98	T_1_ < T_2_⋅T_3_
Physical activity	2.08 (0.96)	2.56 (0.55)	2.69 (0.52)	1.32	13.5	0.000^∗^	0.26	2.27	2.62	T_1_ < T_2_⋅T_3_
Social interaction	1.44 (0.79)	2.23 (0.87)	2.05 (1.07)	1.70	12.7	0.000^∗^	0.25	1.68	2.13	T_1_ < T_2_⋅T_3_
Interest	1.49 (0.60)	2.15 (0.75)	2.10 (0.88)	1.71	12.5	0.000^∗^	0.25	1.74	2.10	T_1_ < T_2_⋅T_3_
Satisfaction	1.26 (0.75)	1.92 (1.01)	1.92 (0.96)	2	16.6	0.000^∗^	0.30	1.45	1.95	T_1_ < T_2_⋅T_3_
Somatic complaints	2.10 (1.20)	2.28 (1.10)	2.38 (1.10)	2	2.5	0.091	0.06	1.91	2.61	
Nervousness	2.64 (0.84)	2.64 (0.74)	2.85 (0.43)	1.84	1.8	0.181	0.04	2.54	2.88	

*p < 0.05, *n* = 39, *M*, mean; *SD*, standard deviation; *df*, degrees of freedom; *F*, Fisher’s *F* ratio, η^2^, effect size.

### Hypothesis 2

Our proposed technological game intervention will have a positive impact on the psychosocial state of caregivers, incrementing the levels of affection, proximity, interest, satisfaction and emotional reaction, and decreasing the levels of nervousness.

The caregiver’s rating results were partially in line with the formulated hypothesis. The analysis of the observational scale scores using RM ANOVA revealed a significant main effect of time, suggesting a significant improvement in the caregiver’s psychosocial factors *affection, proximity, interest*, and *satisfaction* in the 1st and 3rd stages, while there were no significant differences in the rating of these factors in the 2nd stage. On the other hand, *nervousness* and *emotional reactions* did not indicate significant differences between any of the observation stages (see Table 3), nor were significant differences found by age group or sex.

**TABLE 3:** *ANOVA* of psychosocial factors and observation times in caregiver’s ratings.

	Pre playing	10′ playing	20′ playing	Time						Result
					
	*M (SD)*	*M (SD)*	*M (SD)*	*df*	*F*	*p*	η^2^	95% Confidence interval		
									
								Lower bound	Lower bound	
Affection	1.39 (0.79)	2.03 (0.47)	2.03 (0.59)	1.45	14	0.000^∗^	0.30	1.67	1.97	T_1_ < T_2_⋅T_3_
Proximity	1.52 (0.57)	2.06 (0.50)	2.00 (0.61)	2	11	0.000^∗^	0.26	1.73	1.99	T_1_ < T_2_⋅T_3_
Interest	1.39 (0.61)	2.06 (0.56)	1.82 (0.95)	1.63	11	0.000^∗^	0.26	1.56	1.95	T_1_ < T_2_⋅T_3_
Satisfaction	1.42 (0.87)	2.18 (0.68)	2.06 (0.83)	1.77	12	0.000^∗^	0.28	1.68	2.09	T_1_ < T_2_⋅T_3_
Nervousness	2.67 (0.74)	2.76 (0.66)	2.73 (0.72)	2	0.27	0.696	0.01	2.51	2.92	
Emotional reactions	0.82 (1.10)	1.09 (1.30)	0.79 (1.12)	1.67	1.9	0.164	0.06	0.53	1.26	

*p < 0.05, *n* = 39, *M*, mean; *SD*, standard deviation; *df*, degrees of freedom; *F*, Fisher’s *F* ratio; η^2^, effect size.

### Hypothesis 3

The positive psychosocial impact of our proposed technological game intervention will be higher for the patients (mood and depression) and caregivers (mood, depression, anxiety, somatic complaints, and wellbeing) with a psychological negative state (low rating in happiness and wellbeing, and higher rating in sadness, anger, fear, depression, anxiety and somatic complaints).

**FIGURE 5:**
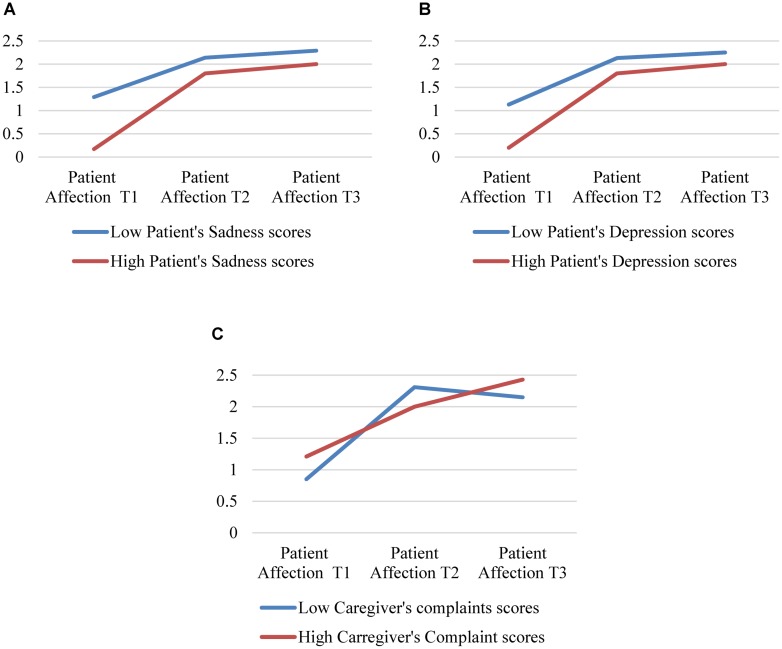
Patient’s affection rating interaction with the patient’s psychological condition. **(A)** Patient’s affection and patient’s sadness, **(B)** Patient’s affection and patient’s depression, **(C)** Patient’s affection and patient’s and caregiver’s complaints.

The results of the interaction analysis were also in line with the formulated hypothesis. The analysis of the psychological test scores and the observational scale scores by ANOVA 2 × 2 revealed a significant interaction effect between the patient’s *affection* rating factor and the patient’s psychological conditions, *sadness, depression* and caregiver’s *somatic complaints* (see Table 4 and Figure 5).

**TABLE 4:** *ANOVA 2* × *2* patient’s affection rating interaction with the patient’s psychological condition.

		Pre playing	10′ playing	20′ playing	Patient affection * Patient’s psychological condition					
						
		*M (SD)*	*M (SD)*	*M (SD)*	*df*	*F*	*p*	η^2^	95% Confidence interval	
								
									Lower bound	Lower bound
Sadness	Low	1.29 (0.75)	2.14 (0.69)	2.29 (0.76)	2	10.2	0.001^∗^	0.48	1.25	1.99
	High	0.17 (0.11)	1.80 (0.40)	2.00 (0.63)						
Depression	Low	1.13 (0.83)	2.13 (0.64)	2.25 (0.71)	2	4.2	0.028^∗^	0.28	1.19	1.97
	High	0.20 (0.45)	1.80 (0.45)	2.00 (0.71)						
Caregiver	Low	0.85 (0.69)	2.31 (0.63)	2.15 (0.55)	2	3.8	0.029^∗^	0.13	1.61	2.06
Complaints	High	1.21 (0.81)	2.00 (0.88)	2.43 (0.65)						

*p < 0.05, *n* = patient conditions groups. Patient with low levels of sadness *n* = 7 and patient with high levels of sadness *n* = 6. Patient with low levels of depression *n* = 8, and patient with high levels of depression *n* = 5. Patient with low levels of somatic caregiver complaints *n* = 13, and patient with high levels of somatic caregiver complaints *n* = 14. *M*, mean; *SD*, standard deviation; *df*, degrees of freedom; *F*, Fisher’s *F* ratio; η^2^, effect size.

**FIGURE 6:**
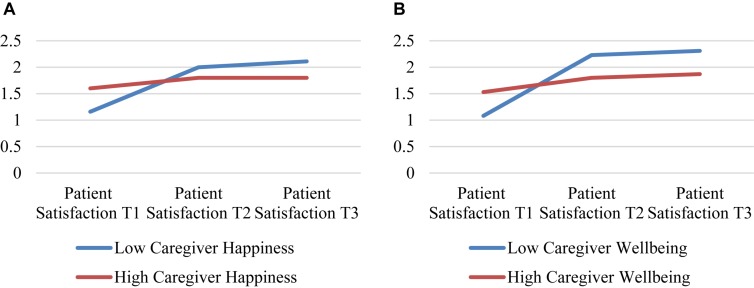
Patient’s satisfaction interaction with the caregiver’s psychological condition. **(A)** Patient’s satisfaction and caregiver’s happiness**, (B)** Patient’s satisfaction and caregiver’s wellbeing.

On the other hand, the other patient’s psychological variables, *happiness, anger*, and *fear* did not show a significant interaction effect. In the case of the caregiver’s psychological state, the analysis also revealed a significant interaction effect between the patient’s *satisfaction* rating factor and the caregiver’s psychological conditions, *happiness* and *wellbeing* (see Table 5 and Figure 6).

**TABLE 5:** *ANOVA 2* × *2* patient’s satisfaction rating interaction with the caregiver’s psychological condition.

		Pre playing	10′ playing	20′ playing	Patient satisfaction * caregiver’s psychological condition					
						
		*M (SD)*	*M (SD)*	*M (SD)*	*df*	*F*	*p*	η^2^	95% Confidence interval	
								
									Lower bound	Lower bound
Caregiver happiness	Low	1.16 (0.76)	2.00 (0.88)	2.11 (0.87)	2	3.19	0.049^∗^	0.106	1.41	2.08
	High	1.60 (0.84)	1.80 (1.30)	1.80 (1.20)						
Caregiver wellbeing	Low	1.08 (0.76)	2.23 (0.92)	2.31 (0.95)	2	6.53	0.003^∗^	0.201	1.50	2.11
	High	1.53 (0.83)	1.80 (1.00)	1.87 (0.91)						

*p < 0.05, *n* = caregiver conditions groups: caregivers with low levels of happiness *n* = 19 and caregivers with high levels of happiness *n* = 10; caregiver with low levels of well-being *n* = 13 and caregivers with high levels of wellbeing *n* = 15. *M*, mean; *SD*, standard deviation; *df*, degrees of freedom; *F*, Fisher’s *F* ratio; η^2^, effect size.

The results showed no significant interaction in terms of interaction with the other psychosocial factors observed in the parents.

### Qualitative Results: Key Themes

**FIGURE 7:**
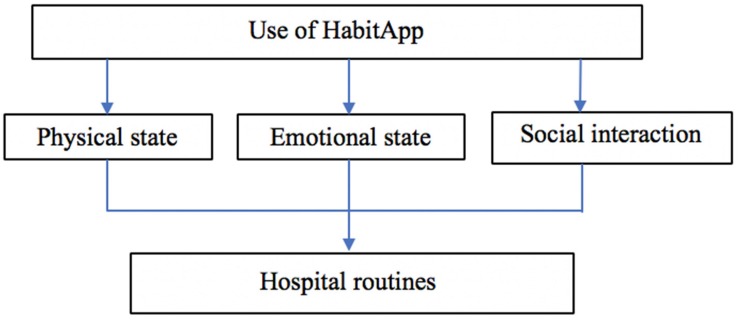
Thematic analysis: significant event of HabitApp experience.

The qualitative results, collected during the observations, provided a set of significant events that defined four important aspects of the use of HabitApp. These themes were: *physical state, emotional state, social interaction*, and *hospital routines* (see Figure 7). Each theme is described in the table below and some real cases are indicated as representative examples of the significant event nature (see Table 6).

**TABLE 6:** Significant event categories and cases.

Thematic analysis	Real case report examples
*Physical state*: the session has a direct or indirect impact on patients’ physical state. *n* = 7 cases The patient shows: - Better resistance to fever [*n* = 3]. - Improved pain control [*n* = 2]. - Increased fine mobility in patients with neurological impairment [*n* = 2].	“Superman, 7 years old, was very ill and has neurological problems that prevent him from moving and speaking normally. HabitApp managed to motivate him to take pictures by working on the fine mobility of his fingers and to try to speak during the session, imitating animal sounds. His doctor and mom were very surprised to see HabitApp encouraging and activating him” “Princess Leia, 10 years old, has undergone numerous surgical operations and was in severe pain during the sessions. The nurses had given her as much pain medication as possible and could not alleviate her. Using HabitApp she managed to relax and was distracted from her pain and also stopped demanding more medication from the nurses” “Cat-woman, 9 years old, had a high fever; the medicine could not get it down and she had been lying in bed all day, while her mother was putting wet towels on her head to try to reduce the fever. She and her mother were very happy when they discovered HabitApp, she got out of bed and started exploring for animals, laughing together with her mother. The fever went down within 10 min after the session started. Mom and nurses were very surprised”
*Emotional state:* the session had a significant impact on mood state, achieving positive states *n* = 10 cases	“Wonder-woman, 6 years old, was feverish and didn’t want to do anything else over several days. Hospital teachers had tried to encourage her through activities but she did not want to do anything. When she discovered HabitApp she asked to have more sessions, she was very interested and smiling and taking many pictures. In addition, after the session she wished to go to the hospital school. Teachers and nurses were surprised” “Iron-man, 12 years old, was very sad, for many days he did not want to do anything, and also was annoyed and irritated for treatment-induced distress. He found the session with HabitApp stimulating and showed expressively positive affection, unlike the previous days. During the session he relaxed and expressed emotions and worries he had experienced during the hospital stay to his caregiver”
*Social interaction:* the session had a significant impact on interpersonal communication. *n* = 14 - Between the patient and caregiver [*n* = 7]. - Between patient and health medical staff [*n* = 7].	“Hulk, 7 years old, used his personal tablet device extensively when he was hospitalized, which alarmed his mother because he was not interested in any other activity and did not have proper communication with anyone. She believed he was addicted to it. When he discovered HabitApp, he stopped using his tablet immediately and began to use the tool, interacting with his mother and constantly talking to her” “Thor, 9 years old, had intense nausea and vomiting caused by the treatment. He did not want to see or talk to anyone at the hospital and had fairly defensive and aggressive behavior. During the HabitApp session, his nausea and vomiting were reduced, rapidly forgotten, and he had a totally different attitude toward the healthcare staff, being rather friendly and polite and responding to nurses’ comments” “Both Deadpool, 5 years old from Algeria and his mother spoke Spanish with difficulty, so they did not have much interaction with the health staff. Using HabitApp, they remembered anecdotes from their country, telling them to the health staff, which created a relaxed and communicative atmosphere. The nurses were surprised by the more communicative and friendly change of attitude”
*Hospital routines*: the sessions had an impact on the performance of daily routines in the hospital. n = 7 Using HabitApp was very useful: - To relax patients during painful or frightening interventions, such as venepuncture [n = 2]. - To promote the intake necessary for their recovery [n = 3]. - To help them take medication [n = 7]. - To motivate them to wake up in the morning as well as to clean themselves [n = 1].	“Lara-croft, 9 years old, was very weak and very affected by the aggressive treatment, had extreme weight loss and developed an aversion to food. Using HabitApp relaxed her and simultaneously helped her to start eating. Doctors reported a significant change in her medical history regarding food” “Cat-woman, 9 years old, was very concerned because she had to go to the operating theater. Her grandmother and her doctor informed us how nervous she was. HabitApp managed to catch her attention and distract her concern. The health team and her caregiver told us that this change in her attitude had been surprising. The caregiver spent much time watching the animals and invited some nurses to observe them” “Batman, 3 years old, was very bored and eager to be alone in the transplant room. His father was exhausted and did not have any ideas to amuse his son. HabitApp was a powerful tool for the father, who got his son into a fun and relaxing playful and educational activity that was interesting for both of us” “Spider-Man, 4 years old, was going home, but first the nurses had to remove the port-a-cath. Spiderman was really nervous and crying. Watching giraffes on HabitApp managed to reduce his crying, and decrease his anxiety while the nurses did the intervention”
*N* total = 37 cases of significant events	

### User Experience Results

**FIGURE 8:**
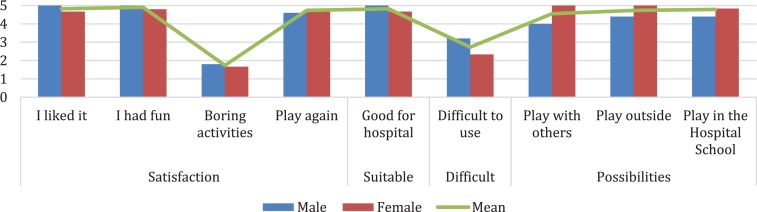
Patient’s HabitApp User Experience. Rating score: 1 = nothing, 2 = little, 3 = something, 4 = quite a lot, 5 = very much.

HabitApp user experience was evaluated thought a Likert questionnaire. The results indicated good patient *satisfaction* (items 1, 4, 5, and 6) with an average of 3.18 points. They also considered it an *adequate* tool for the hospital context (item 2) with an average of 4.82 points. On the other hand, the perceived *difficulty* of use (items 3) was given 2.73 points on average and the *potential* for improvement (items 7, 8, 9) of HabitApp 4.68 points (see Figure 8).

**FIGURE 9:**
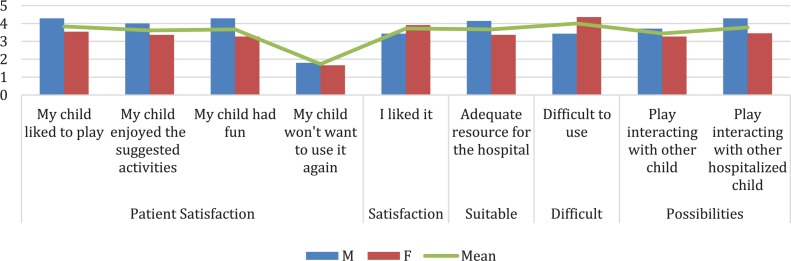
Caregiver’s HabitApp User Experience. Rating score: 1 = strongly disagree, 2 = disagree, 3 = undecided, 4 = agree, 5 = strongly agree.

The caregivers’ user experience indicated an average of 3.7 points for the satisfaction of their child with HabitApp (items 1, 4, 5, and 6) and an average of 3.72 points for their own satisfaction (item 8). The adequacy of HabitApp for the hospital context (item 2) obtained an average of 3.67 points. The perceived difficulty of use (item 3) had an average of 4 points and Habitat’s potential for improvement (item 7) had an average of 3.6 points (see Figure 9).

## Discussion

The present study proposes the inclusion of a technological infrastructure in the context of childhood cancer, enabling collaborative animal observation activities in which both patient and caregivers can participate. The review of the related works revealed a lack of studies that propose the type of approach used in HabitApp, and also the lack of works that evaluate the psychosocial impact of game interventions on both patients and caregivers. The HabitApp mobile application allows a remote connection and interaction with the natural environment outside the hospital. Users are able to connect in real time and interact with remotely controlled cameras at a local zoo, promoting interaction between patients and caregivers. The characteristics of HabitApp are important, given the known benefits of being in contact with nature and animal assisted intervention (Fine and Weaver, 2018) to people’s health, especially in the isolation context of a pediatric oncology unit. These conditions are especially strict in patients that have undergone bone marrow transplants and most of them could never benefit from interventions involving contact with natural environments and animals. This type of technology has given these patients the possibility of interacting with nature and animals while hospitalized in isolation units.

HabitApp’s main research results are therefore in terms of psychosocial factors and a positive impact on hospitalized patients and caregivers. The analysis of the psychosocial impact has demonstrated that HabitApp evoked expressions and behavior that denote an increase of positive affection, with smiling faces and laughter and interaction between patients and caregivers, promoting relaxed conversations, storytelling, and increased collaboration. In addition, many of the caregivers were physically closer and showed more affection to the patients during the intervention.

At the same time, HabitApp succeeded in engaging the user’s attention, showing an active exploration of the application’s functionalities and innovative thinking about improvements to the existing functionalities. Finally, both patients and caregivers reported satisfaction and enjoyment in the user experience questionnaires and supported by the qualitative data, with comments such as “what fun!”, “I like it a lot!”, “I am having a really good time!”, highlighting the power of new technologies to serve a very wide age range (Duffy et al., 2005).

It should be noted that the results were obtained with 10-min sessions after 20 min of play, which is very beneficial, given the clinical conditions of the patients, who are subjected to numerous interventions by health personnel and generally do not have much time for play.

Another key point of our study was the psychological state of patients and caregivers, because both experience negative emotions from hospitalization and treatments (Schultz et al., 2007).

The *post hoc* analysis of the psychological state indicated that the children with highest levels of depression obtained most benefit from the positive effects. Similarly, HabitApp achieved a greater increase in positive emotions in children whose parents had fewer somatic complaints; although the tool can positively affect children’s emotions, it was not as effective in those whose parents showed somatic symptoms of stress (Wechsler et al., 2014). These results supports the emotional transfer theory between patients and caregivers during hospitalization, which affects the way in which patients react to interventions in the hospital according to their parents’ emotional state (Fernández-Castillo and López-Naranjo, 2006).

In spite of this, the children’s capacity for enjoyment during the intervention did not seem to be affected by the negative emotional state of the caregivers, since the children whose parents showed the most negative emotions were the ones who expressed the greatest satisfaction with the game, so that it could be said that HabitApp met one of the main objectives of play therapy, to maximize the enjoyment of life.

Our results thus show that technological game interventions, including multidisciplinary interventions, could form part of an integral approach to this pathology. They also reveal the need to integrate play for therapeutic purposes for both patients and their caregivers (Montoya and Barrón, 2001). With this aim, medical staff, psychologists and technological game designers should focus on fostering positive emotions and reducing stress to improve the patients and caregiver’s hospitalization experience and achieve significant results.

Our future line of work will use the study results to improve HabitApp’s design, usability and user experience by including features such as having more cameras available at the zoo or in other natural places. Gamification strategies could also be included in some of the activities, such as taking pictures, sharing them and making albums.

Our work has some limitations that have to be considered. Our work evaluates the short-term impact of HabitApp during 3 weeks of use. However, it would be interesting to consider its long-term impact in order to evaluate its ability to reduce the days of hospitalization or increase the effectiveness of the clinical interventions. A long-term examination, increasing the sample size, would allow us to evaluate the impact of age, gender or clinical condition. Despite this limitation, the short-term impact of game interventions in the context of oncology pediatric patients who are in isolation is still of great value in situations in which clinical staff need a fast short-term psychological effect (before/after clinical invasive procedures, with negative psychological states related to unforeseen clinical events, etc.). Another limitation of this work is the lack of a control group (not exposed to HabitApp) that would have allowed us to directly compare two samples of patients. However, the low number of available patients (available clinical population) in the Hemato-oncological Unit of La Fe Hospital (despite being the largest hospital in the Valencia Region) prevented us from having this experimental design. Because of logistic and economic limitations we had no means to run this evaluation in several hospitals in Spain and we opted for an experimental design in which a single group of 39 patients and caregivers were evaluated in the use of HabitApp to get the maximum possible statistical power with the available clinical population. This should be considered in the future to improve the external validity of our work. The internal validity could possibly be improved by a second evaluation of psychological states to indicate not only whether they affect the tool’s impact, but also whether they are affected by the tool itself, assessing the participant psychological state repeatedly during the hospitalization. This was a limitation in our study because some of the patients were discharged or their clinical condition worsened and obtaining multiple evaluations with a larger number of participants was not possible with the current resources for this study.

## Conclusion

Despite the previous limitations, it can be said that the study confirms HabitApp as an effective collaborative psychosocial intervention tool that improves the hospitalization experience in the short-term for patients and caregivers in the context of a children’s cancer ward.

## Data Availability Statement

All datasets generated for this study are included in the article/Supplementary Material.

## Ethics Statement

This study was carried out in accordance with the recommendations of the Hospital La Fe of Valencia ethics committee with written informed consent from all subjects. All subjects gave written informed consent in accordance with the Declaration of Helsinki. The protocol was approved by the Hospital La Fe of Valencia ethics committee.

## Author Contributions

IM-C and JJ conceived the work. All authors participated in the design of the study, interpretation of the results, contributed significantly to its critical review in later versions, and approved the final version of the manuscript. AC-P did the field work and wrote the first draft of the manuscript.

## Conflict of Interest

The authors declare that the research was conducted in the absence of any commercial or financial relationships that could be construed as a potential conflict of interest.
