# Supplementary material for: HabitApp: New Play Technologies in Pediatric Cancer to Improve the Psychosocial State of Patients and Caregivers
Source: Front Psychol. 2020 Feb 7;11:157. doi: 10.3389/fpsyg.2020.00157 (PMC7020696; doi:10.3389/fpsyg.2020.00157)
Supplement: Supplementary file 1 [file Data_Sheet_1.pdf]

S1. Observational Scale

	Traits	Observational Evaluation	Comments
1.	Affection: Facial and body expression denoting a type of emotion (positive and negative).	0: Serious, sad or bored face.<br>1: There is no emotional expression, flat affect.<br>2: Smile, happy or cheerful face.<br>3: Enthusiasm or surprise (positive) face, roars of laughter.	"…"
2.	Nervousness: repetitive movements (i.e. tics), verbalizations about worries or fears, anxious attachment.	0: More than two nerve samples.<br>1: Two samples of nerves.<br>2: One sample of nerves.<br>3: No nerve samples.	
3.	Interests: attention to the tool and its different functionalities.	0: does not pay attention, does not want to use it.<br>1: he uses it but in a passive way without paying much attention.<br>2: Shows interest, actively uses it exploring the different functionalities.<br>3: Shows a lot of interest, uses it proactively, proposing new functionalities.	
4.	Satisfaction: comments about the experience using the tool.	0: At least one negative comment, such as, "this is boring", "I don't like it".<br>1: No comment.<br>2: A positive comment, such as, "this is amusing", " I like a lot", " I have fun".<br>3: More than one positive comment.	
5.	Somatic Complaints: verbal	0: More than two complaints or	

	expressions of pain, grimaces of pain, touching an affected part of the body with signs of discomfort.	signs.<br>1: Two complaints or signs.<br>2: One complaint or sign.<br>3: No complaint.
6.	Physical Activity: movements related to the child's physical condition.	0: No movement.<br>1: Almost no movement.<br>2: Shows movement.<br>3: A lot of movement.<br>* In children with physical limitations: has limb movement, raises arms, incorporates himself.
7.	Social interaction: individual use, managed interaction, interaction management, collaboration play.	0: Individual play.<br>1: Child responds to another person's interaction.<br>2: Child addresses the interaction with another person.<br>3: Collaborative play.
8.	Proximity: caregiver's position with respect to the child'	0: Not with the patient.<br>1: Separated from the patient (no interaction).<br>2: Close to the patient, low level of interaction with each other)<br>3: In physical contact with the patient, high level of interaction.
9.	Emotional Reaction: verbal responses to emotional reactions.	0: Reject the child's emotional reactions. Towards positive emotions use comments such as "now it doesn't hurt", "how happy you are now, with what you were complaining about before" (ironic tone). Towards negative emotions, "if you cry, you won't keep playing",

	"don't shout".<br><br>1: There are no emotional reactions to the child's emotions, or the child does not show a certain emotion.<br><br>2: Allows emotional reactions of the child without offering feedback.<br><br>3: Validates the child's emotional reactions and provides adequate feedback to positive coping models. Express understanding and empathy with the child with expressions such as "have a good time", "we are having a great time", "it is normal for you to feel pain", "you are right, it should hurt a lot", "you are very strong".

Note: 1 - 4 assessed patients and caregiver's behaviour, 5 – 7 assessed just patient's behaviour; and 8 and 9 assessed just caregiver's behaviour.
