# Supplementary material for: HabitApp: New Play Technologies in Pediatric Cancer to Improve the Psychosocial State of Patients and Caregivers
Source: Front Psychol. 2020 Feb 7;11:157. doi: 10.3389/fpsyg.2020.00157 (PMC7020696; doi:10.3389/fpsyg.2020.00157)
Supplement: Supplementary file 2 [file Data_Sheet_2.pdf]

S2. User experience questionnaires

Patient/ User Experience questionnaire

We would like to know if you have enjoyed using HabitApp. Please answer the following questions.

	nothing	little	something	quite a lot	very much
1. I liked to use it.	○	○	○	○	○
2. I think it is a good technology/game tool for the hospital.	○	○	○	○	○
3. I think it is difficult to use.	○	○	○	○	○
4. I had fun.	○	○	○	○	○
5. The activities (taking pictures, sharing them) were boring.	○	○	○	○	○
6. I would like to play again.	○	○	○	○	○
7. I would like to play again with another child.	○	○	○	○	○
8. I would like to play with the app outside of the hospital.					
9. I would like to play with the app in the hospital school.					

Caregivers/ User Experience questionnaire

We would like to know your comments about the HabitApp tool. Please answer the following questions.

	strongly disagree	disagree	undecided	agree	strongly agree
1. My child liked using the app.	○	○	○	○	○
2. I think this is an appropriate technological resource to improve the interaction with my child.	○	○	○	○	○
3. I think that the app is hard to use for my child.	○	○	○	○	○
4. I think my child enjoyed watching the animals in the app.	○	○	○	○	○
5. I think my child had fun taking pictures with the app.	○	○	○	○	○
6. I think my child will not want to use the app again.	○	○	○	○	○
7. I think my child would like to use the app interacting with another child.	○	○	○	○	○
8. I liked using the app.	○	○	○	○	○
9. I think my child would like to use the app interacting with another hospitalize child.	○	○	○	○	○
